# 549 Perioperative Complications in Burn Surgery Procedures in a Single ABA Verified Burn Center

**DOI:** 10.1093/jbcr/iraf019.178

**Published:** 2025-04-01

**Authors:** Curtis Swanson, Kaitlyn Libraro, Abraham Houng

**Affiliations:** Weill Cornell Medical College; Weill Cornell Medical College; New York Presbyterian Hospital - Weill Cornell

## Abstract

**Introduction:**

Burn surgery is needed for wound closure, restoration of function, and improvement of cosmesis. However, it is not without risk. There are several known complications regarding burn surgery: bleeding, infection, and graft loss. The rate of perioperative complications range from 0.3% to greater than 20% in the literature. Through our burn center’s quality improvement program, we examined our institution’s perioperative complication rate in a given year, and were able to provide an updated rate of perioperative complications for burn surgery.

**Methods:**

Data from weekly quality improvement meeting was used to capture the following perioperative complications from burn surgery procedures in the operating room: autograft loss requiring subsequent procedures, bleeding, infection, donor site failure, and intra-operative complications. Time ranged from 1/1/2023 to 12/31/2023. Data abstracted included demographics, medical history, injury details, and surgical procedure.

**Results:**

In 2023, we performed 317 burn operations. This included 193 autograft, 86 allograft, 17 skin substitutes, and 21 excision/debridement without grafting. In the autograft group, there were 3 autograft loss requiring subsequent procedures (1.6%), 3 post-op bleeding (1.6%), 2 donor site failure (1.0%), and 3 intra-operative complications (1.6%). In the skin substitute group, there were 2 post-op bleeding (11.8%), 2 infections with bacterial organism (11.8%), and 2 skin substitute loss (11.8%). There were no reported complications in the allograft and procedures without grafting groups.

**Conclusions:**

In this retrospective review, most of the complications noted were in patients with deep third degree burn or burns with loss of underlying structure. Skin substitute complication is much higher than autografting because skin substitute was mostly used in cases where auto/allografting is not possible or in cases with non-vascularized structures such as bone or tendon. Perioperative complication in burn surgery procedures has not been widely published, and published results vary in order of magnitude. We are providing an updated data on perioperative complications; so we may use this data to better counsel patients who will be undergoing burn procedures in the future.

**Applicability of Research to Practice:**

Directly applicable

**Funding for the Study:**

N/A

